# 

**Published:** 1899-03

**Authors:** 


### BOOKS RECEIVED.

Three Thousand Questions on Medical Subjects. Arranged for self-examination, with the proper references to standard works in which the correct replies will be found. Second edition, enlarged. Philadelphia: P. Blakiston's Son & Co. 1899.

An Experimental Research into Surgical Shock. An essay awarded the Cartwright prize for 1897. By George W. Crile, A. M., M. D., Ph. D., Professor of Surgery and Applied Anatomy in the Cleveland College of Physicians and Surgeons; formerly Professor of Physiology in the Medical Department, University of Wooster; Attending Surgeon to the St. Alexis and Cleveland General Hospitals. Philadelphia: J. B. Lippincott Company. 1899.

A Treatise on Fractures and Dislocations. For Practitioners and Students. By Lewis A. Stimson, B. A., M. D., Professor of Surgery in Cornell University Medical College, New York. In one 8vo volume of 823 pages, with 321 engravings and 20 full-page plates. Cloth, $5.00 net; leather, $6.00 net. Just ready. Philadelphia and New York: Lea Brothers & Co.

Railway Surgery. A Handbook on the Management of Injuries. By Clinton B. Herrick, M. D., Troy, N. Y., Lecturer in Clinical Surgery, Albany Medical College; Attending Surgeon to the Troy Hospital and the House of Good Shepherd; Consulting Surgeon to the Leonard Hospital; Surgeon to the Delaware & Hudson and the Fitchburg Railways; President of the New York State Association of Railway Surgeons. New York: William Wood & Co. 1899.

Transactions of the Medical Society of the State of North Carolina. Forty-fifth Annual Meeting, held at Charlotte, May 3, 4, and 5, 1898. Winston: Carolina Publishing Company. 1898.

Fever Nursing. Designed for the Use of Professional and Other Nurses, and Especially as a Text-book for Nurses in Training. By J. C. Wilson, A. M., M. D., Professor of the Practice of Medicine and of Clinical Medicine in the

Jefferson Medical College; Visiting Physician to the Hospital of the Jefferson College and the Pennsylvania Hospital; Physician-in-Chief to the German Hospital, etc., etc. Third edition, revised and enlarged. Philadelphia: J. B. Lippincott Company. 1899.

Transactions of the College of Physicians of Philadelphia. Third series, Vol. XX. Philadelphia: William J. Dornan, Printer. 1898.

Diseases of the Eye. A Handbook of Ophthalmic Practice for Students and Practitioners. By G. E. de Schweinitz, A. M., M. D., Professor of Ophthalmology in the Jefferson Medical College; Professor of the Diseases of the Eye in the Philadelphia Polyclinic; Ophthalmic Surgeon to the Philadelphia Hospital, etc., etc. With 255 illustrations and two chromo-lithographic plates. Third edition, thoroughly revised; 8vo, pp. 699. Philadelphia: W. B. Saunders. 1899.

An American Text-book of Diseases of the Eye, Ear, Nose and Throat. Edited by G. E. de Schweinitz, A. M., M. D., Professor of Ophthalmology in the Jefferson Medical College, Philadelphia; Consulting Ophthalmologist to the Philadelphia Polyclinic; Ophthalmic Surgeon to the Philadelphia Hospital and to the Orthopedic Hospital and Infirmary for Nervous Diseases; and B. Alex. Randall, M. A., M. D., Ph. D., Clinical Professor of Diseases of the Ear in the University of Pennsylvania; Professor of Diseases of the Ear in the Philadelphia Polyclinic; Ophthalmic and Aural Surgeon to the Methodist and Children's Hospitals. Illustrated with 766 engravings, 59 of them in colors. Imperial 8vo, pp. 1251. Philadelphia: W. B. Saunders. 1899.

